# Promoting cognitive health: a virtual group intervention for community-living older adults

**DOI:** 10.1590/1980-5764-DN-2022-0020

**Published:** 2023-05-05

**Authors:** Tamires Nicodemos Vasques, Maria Helena Morgani de Almeida, Rosé Colom Toldrá, Marina Picazzio Perez Batista

**Affiliations:** 1 Universidade de São Paulo, Faculdade de Medicina, Departamento de Fisioterapia, Fonoaudiologia e Terapia Ocupacional, São Paulo SP, Brazil.

**Keywords:** Aged, Cognition, Remote Consultation, COVID-19, Idoso, Cognição, Consulta Remota, COVID-19

## Abstract

Group cognitive interventions can promote a sense of self-efficacy to older adults. Due to restrictive social distancing measures in the COVID-19 pandemic, face-to-face interventions that aimed to promote cognitive health needed to be adapted to a virtual offering.

**Objectives:**

This study aimed to analyze the effects of promoting cognitive health in a virtual group intervention for community-living older adults.

**Methods:**

This is a mixed, prospective, and analytical study. Before and after the intervention, the tests were applied: Brief Cognitive Screening Battery (BCSB) and the Subjective Memory Complaints Questionnaire (MAC-Q). Data were collected at semi-structured interviews related to the adoption of memory strategies. Statistical tests were conducted for initial and final intragroup comparison. The qualitative data were assessed using thematic analysis.

**Results:**

A total of 14 participants concluded the intervention. With respect to mnemonic strategies, the most relevant for the qualifier “Did not use it before and started to do so after the group” were association (n=10; 71.4%) and dual-task inhibition (n=9; 64.3%). According to the tests, the intervention improved incidental, immediate, and delayed recall, as well as the perception of memory for “Remembering the name of the person they just met,” “Remembering the telephone number you use at least once a week,” “Remembering where they put an object,” “Remembering news from a magazine article or television program,” and “In general, how would you describe your memory now compared to when you were 40 years old.”

**Conclusions:**

The synchronous virtual group intervention was shown to be feasible for the elderly in the community who participated in the study.

## RESUMO

Intervenções cognitivas em grupo podem promover um senso de autoeficácia em idosos. Em decorrência das medidas restritivas de distanciamento social na pandemia de COVID-19, as intervenções presenciais que visavam promover a saúde cognitiva precisaram ser adaptadas à oferta virtual.

**Objetivos:**

Analisar os efeitos da promoção da saúde cognitiva em uma intervenção em grupo virtual para idosos que vivem na comunidade.

**Métodos:**

Trata-se de um estudo misto, prospectivo e analítico. Antes e depois da intervenção foram aplicados os testes: Bateria de Triagem Cognitiva Breve (BCSB) e Questionário de Queixas de Memória Subjetiva (MAC-Q). Os dados foram coletados em entrevistas semiestruturadas relacionadas à adoção de estratégias de memória. Foram realizados testes estatísticos para a comparação intragrupo inicial e final. Os dados qualitativos foram avaliados por meio da análise temática.

**Resultados:**

Quatorze participantes concluíram a intervenção. Com relação às estratégias mnemônicas, as mais relevantes para o qualificador “não usava antes e passou a usar depois do grupo” foram associação (n=10; 71,4%) e inibição de dupla tarefa (n=9; 64,3%). De acordo com os testes, a intervenção melhorou a recordação incidental, imediata e tardia, bem como a percepção da memória para: “lembrar o nome da pessoa que acabou de conhecer”; “lembrar o número de telefone que você usa pelo menos uma vez por semana”; “lembrar onde colocaram um objeto”; “lembrar notícias de um artigo de revista ou programa de televisão” e “em geral, como você descreveria sua memória agora em comparação com quando tinha 40 anos”.

**Conclusões:**

A intervenção síncrona em grupo virtual mostrou-se viável para os idosos da comunidade que participaram do estudo.

## INTRODUCTION

Projection data for the Brazilian population until 2060 estimate that the number of elderly people will more than double, from 10.5 to 25.5% of the total population^1^. This finding implies the definition of measures to age with quality of life supported for health policies^2^. Studies reveal that even the expected aging process is permeated by the decline of cognitive functions that interfere in the psychological well-being^3^.

The most affected cognitive functions are working memory and executive functions^4^. These changes are gradual and expressed when the elderly person has difficulty combining new information with long-term memory, controlling attention, and responding effectively to complex problems^4,5^. Evidence from randomized studies proves that the promotion of cognitive health is capable of increasing the cognitive reserve of the elderly, suggesting that even neuroanatomical differences can be influenced, to some extent, by environmental factors^6,7^.

Interventions for this purpose include education groups, which focus on changes in lifestyles, health behaviors, and memory strategies that favor the feeling of self-efficacy during aging and improve self-assessed cognitive performance^6,8,9^. Because they help people develop, recover, improve, and maintain the skills needed for daily living and working, occupational therapists are considered best suited to conducting these interventions^9^. Recognizing these benefits and being guided by principles that govern the promotion of health for the elderly, including their mental, physical, and social stimulation^2,10^, since 2005, the Gerontology Laboratory of the Occupational Therapy Course, Faculdade de Medicina da Universidade de São Paulo (University of São Paulo School of Medicine), has offered in-person cognitive intervention group for the community-living older adults^11^.

However, it is necessary to rethink how to follow up the group during the COVID-19 pandemic^12^, where sanitary measures have been adopted to mitigate the disease, resulting in restricted social contact and interfering directly in the routine of the older population and their access to health care^13^. Studies reveal that the main consequences are difficulty concentrating, memory loss, stress, anxiety, depressed mood, and sleep problems^14,15^. To minimize these losses caused by the pandemic, remote consultations started to be offered virtually under the approval of professional health councils^16^.

The benefits of providing online interventions to older adults include cost-effectiveness, convenience, greater access to health information for those with reduced mobility, transportation problems associated with financial limitations, connectivity and social support, and more opportunities for health education^17^.

However, with respect to specific occupational therapy actions in telehealth, one study found that a minority were aimed at cognitive^18^. Considering the relevance of acting on cognitive functions affected by the aging process, especially in the pandemic context, and of expanding evidence on the benefits of group cognitive interventions in the virtual modality, this study aimed to describe and analyze the effects of promoting cognitive health in a virtual group intervention for community-living older adults.

## METHODS

This is a mixed, prospective, and analytical study. The project received approval from the Research Ethics Committee of the Faculdade de Medicina da Universidade de São Paulo (University of São Paulo School of Medicine), under protocol number 06339512.0.0000.0065, on May 26, 2021, and satisfied all ethical standards and demands.

### Participants

Community-living older adults were recruited to participate in remote (synchronous) cognitive intervention between September and December 2021. The proposal was disseminated using the snowball sampling technique^19^, via online groups previously contacted by the researchers. Interested persons filled out a form on Google Forms. In this form, the interested person had to put their phone and e-mail contact and to self-declared as being cognitively healthy and with no diagnosis of neurocognitive or mental disorder. Also, the person had to confirm that they had the technology required to participate in the group.

The first 20 older adults who filled the form were phone contacted to schedule an interview. On this occasion, the researcher confirmed if the person met inclusion criteria for the study. The inclusion criteria were as follows:

- Being 60 years of age or older;
- Living in the community;
- Defined themselves as being cognitively healthy;
- Do not have a diagnosis of neurocognitive or mental disorder;
- Do not have specific cognitive monitoring by health professionals;
- Do not present conditions that preclude virtual interview or group participation, such as severe communication difficulties like aphasia, sensory impairment like deafness, or others severe impairment; and
- Have access to a computer or cell phone equipped with a camera and microphone connected to the WhatsApp and Google Meet platform.

As all the 20 older adults contacted met the inclusion criteria, they were invited to participate in the study. They were divided into two groups, with 10 people each, distributed according to the “Google Forms” filling list order. Each group was led by a resident speech therapist, a physical therapist, and an occupational therapist from the FMUSP Multiprofessional Residency Program for the Promotion and Care of Hospital Health (area: Adult and Elderly Health). Although the two groups were conducted separately, both participated in a standardized intervention. The justification to divide the group in two was to facilitate the older adults participation, as it would be difficult to manage a virtual group with 20 people. The two standardized groups were simultaneously conducted.

### Instruments

The following standardized instruments were used by the same examiner to collect data before and after the intervention:

- Brief Cognitive Screening Battery (BCSB) to assess the cognitive functions of language. This test involves the following domains: Visual Perception and Naming related to incidental recall, immediate recall 1, learning (immediate recall 2), and delayed recall (5 min) and recognition of 10 common black and white drawings with a maximum recall time of 60 s; Verbal Fluency Test of the number of animals recalled in 1 min and the Clock Drawing Test. The total score is the sum of each domain, that is, for the incidental, immediate, and delayed recall, the number of correct answers (0–10); for the recognition domain, the final score is the difference between correct and incorrect answers; in the clock drawing design, the score varies between 6 and 10 points for correct numbers and 1 and 5 for the clock and incorrect numbers^20,21^;
- Subjective Memory Complaints Questionnaire (MAC-Q) to assess subjective memory complaints. Based on six questions, subjects are asked to compare their current memory with their memory at 40 years of age in terms of:
  - Remembering the names of people they just met;
  - Remembering a phone number used at least once a week;
  - Remembering where they put objects;
  - Remembering news from a magazine article or television program;
  - Remembering things that they intend to buy when they arrive at a store, and
  - In general, how would you describe your memory now compared to when you were 40 years old? Each question has five possible answers on a 5-point Likert scale, where the last question is worth double the corresponding value. The total score varies from 7 to 35 points, and the lower the score, the better the perceived memory^22^.

A semi-structured interview was applied to collect sociodemographic data, impact of memory and attention on the daily life of the elderly, the use of strategies to memorize information, familiarity with technologies, and possible difficulties of older individuals in handling them. At the end of the intervention, the group was submitted to a semi-structured interview to obtain the opinion of participants regarding their participation in the group, suggestions for improving the proposal, and possible group benefits with promoting cognitive health. Participants were asked to give their opinion of the strategies used in the group among the following alternatives:

- Did not use it before but started to do so after the group;
- Already used it and started to use it more after the group;
- Did not use it before and continued not using it after the group;
- Already used it and continued using it to the same extent after the group; and
- Already used it and stopped doing so after the group. In addition to the data obtained from assessments and reassessments, data were collected from the researcher’s field diary.

### Procedures

**Figure 1:**
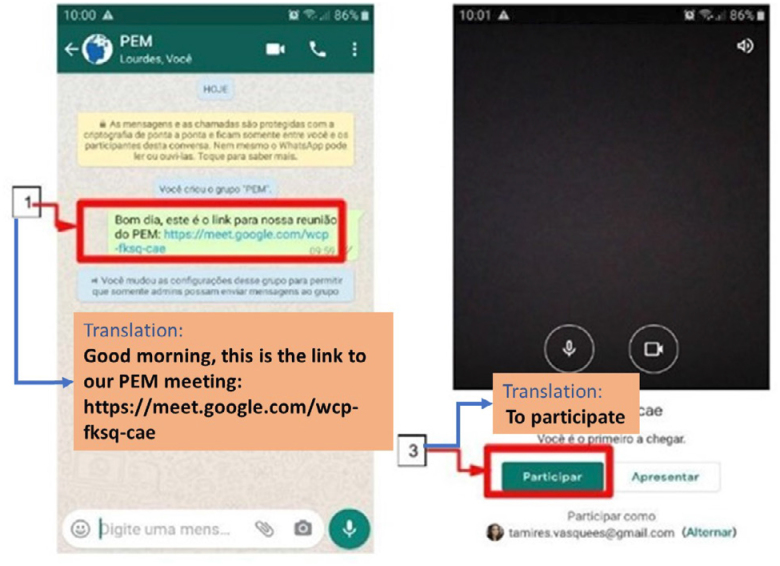
Step by step to connect to video call through Google Meet via WhatsApp link. (1) On the WhatsApp conversation screen, tap the blue link; (2) Wait for the link to load; (3) Tap “Join” and make sure the camera and audio are on.

**Figure 2:**
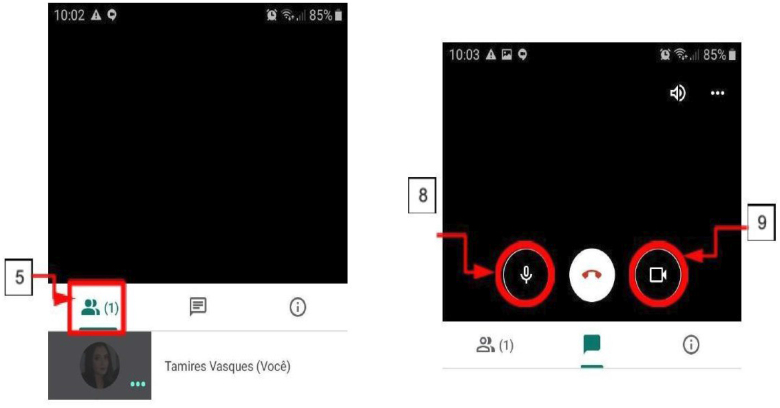
How to identify icons on the Google Meet platform. (4) With the meeting open, you hear and see everyone who has their camera and microphone on; (5) To see the meeting participants, just tap the first icon; (6) To avoid interference, it is recommended to turn off the microphone when other people are talking; (7) Just touch the screen to show the microphone and camera icons, touch each one if you want to turn it off; (8) Microphone icon; (9) Camera icon.

Nine 2 h weekly meetings were held. The following issues were discussed: the concept of memory, mental functions, emotional aspects and memory, memory and aging, lifestyle, and memory and strategies to maintain and improve memory^11^. The description of the activities carried out in each session is described in Chart-1. Google Meet was used to develop the intervention, and a WhatsApp group was created to send reminders and theoretical-practical materials. The subjects who reported difficulty in handling the technology received a tutorial on how to access and use these resources (Figures 1 and 2).

**Chart 1:** Description of the activities carried out in each session. São Paulo, 2021.

Theme at the meeting	Contents
Session 01 Dynamics of presentation Focus on memory	“Affective memory wall” which enabled the participants to present themselves from a photo of what they represent in sense and meaning. Memory concept and functions. The three phases for memory retention were addressed (capture, storage, and recall). Importance of Intention to learn and the meaning of information. Strategies to compensate for impaired senses (glasses, hearing aid), environment (lighting, low noise), and behavioral changes (use of more than one sense, use more keen sense, better times to perform activities).
Session 02 Types of memory	Explicit/declarative memory, short-term and long-term memory. Content-retrieval activities: Who am I?, Popular sayings and What kind of memory is this?
Session 03 Types of attention	Attention: selective, alternating, and divided. Content-retrieval activities: Looks like but isn’t/Figure-background, Lynx eyes game, and Senior dance.
Session 04 Demystifying aging	Cognitive aging. Content-retrieval activities: Myth or truth; Reflection on “Age and Change” and “Utopia of the Perfect Age”.
Session 05 Lifestyle and memory	Diet balanced, practice of physical and mental activities, combating stress, controlling diseases and their relationship with memory. Content-retrieval activities: 21-Day Theory (Building Habits).
Session 06 e 07 Strategies for memory	Association; dual-task inhibition; increased attention; multiple coding; categorization; external device; environmental change and planning; emotion attribution; change in lifestyle; repetition; change in routine and behavior. Content-retrieval activities: memory palace.
Session 08 Memory and emotion	Emotion interferes with capture, storage, and evocation. Storage strategy: attribution of emotion to information, assigns a prominent place in memory. Content-retrieval activities: sensory experiences.
Session 09 Content review	Rescue of main contents, resolution of doubts, closing dynamics, and speech space for suggestions.

### Data analysis

The data of the two groups were organized and analyzed as a single intervention, as they were equally conducted. Subjects were assessed together, consisting of the paired dependent samples of this study. For this purpose, the Statistical Package for the Social Sciences (SPSS) version 2.0 software was used. The study did not have a control group or different interventions groups. In this sense, the data analysis was conducted with the same 20 older adults assessed before and after the intervention, considered together as a single group to be analyzed.

For the analysis obtained from the standardized instruments applied before and after the intervention, the Shapiro-Wilk test was used to determine data normality. For data with non-normal distribution, Wilcoxon’s nonparametric test for paired samples was used for initial and final intragroup comparison of the items and total score in all the instruments. Non-normally distributed data were presented via the median and interquartile range, frequently used in nonparametric tests. The paired t-test was applied for normal distribution, with data presented as the mean and standard deviation. A 5% significance level was established for all the analyses (p<0.05).

Data obtained from the semi-structured interviews applied at reassessment, related specifically to the use of mnemonic strategies and demographic data, were described by simple frequency. The qualitative data from the semi-structured interviews for assessment and reassessment, and from the researcher’s field diary, underwent thematic analysis^23^.

## RESULTS

Of the 20 participants enrolled simultaneously in the two groups, 14 (66.6%) concluded the intervention in terms of frequency and participation in the final reassessment. The characterization of the sample of elderly people who participated in the study can be seen in Table 1. It is observed that 100% (n=14) of the participants who completed the proposal were female, 92.9% (n=13) had completed higher education, 64.2% (n=9) were between 60 and 69 years old, and 35.7% (n=5) needed the tutorial to access digital inclusion platforms.

**Table 1:** Simple descriptive frequency according to sex, age, schooling, and access to technologies. São Paulo, 2021.

Variables		n	%
Sex	Female	14	100
	Male	0	0
Age (years)	60–69	9	64.2
	70–79	4	28.5
	80 years or older	1	7.1
Schooling	Incomplete secondary	1	7.1
	University graduate	13	92.9
Access to Google Meet	Required access tutorial	5	35.7
	Refused to need the access tutorial	9	64.2
	Total	14	100

With respect to mnemonic strategies, the group had a positive influence on their daily use. Table 2 shows that the most relevant for the qualifier “Did not use it before and started to do so after the group” were association (n=10; 71.4%), dual-task inhibition (n=9; 64.3%), increased attention (n=8; 57.1%), multiple coding (n=6; 42.9%), and categorization (n=6; 42.9%).

**Table 2:** Results of adopting mnemonic strategies before and after the intervention. São Paulo, 2021.

Memory strategy	Did not use it before and started to do so after the group (%)	Already used it and started to use it more after the group (%)	Did not use it before and continued not using it after the group (%)	Already used it and after the group continued using it to the same extent (%)
Association	10 (71.4)	3 (21.4)	0	1 (7.1)
Dual-task inhibition	9 (64.3)	2 (14.3)	3 (21.4)	0
Increased attention	8 (57.1)	3 (21.4)	2 (14.3)	1 (7.1)
Multiple coding	6 (42.9)	5 (35.7)	1 (7.1)	2 (14.3)
Categorization	6 (42.9)	3 (21.4)	0	5 (35.7)
External device	1 (7.1)	8 (57.1)	0	5 (35.7)
Environmental change and planning	1 (7.1)	5 (35.7)	2 (14.3)	6 (42.9)
Emotion attribution	3 (21.4)	6 (42.9)	4 (28.6)	1 (7.1)
Change in lifestyle	2 (14.3)	6 (42.9)	1 (7.1)	5 (35.7)
Repetition	3 (21.4)	4 (28.6)	5 (35.7)	2 (14.3)
Change in routine and behavior	1 (7.1)	5 (35.7)	3 (21.4)	5 (35.7)

The cognitive performance data according to the BCSB score before and after the intervention are presented in Table 3. The individual analysis of the instrument items shows that incidental, immediate, and delayed recall improved with the intervention.

**Table 3:** Results of the BCSB before and after the intervention. São Paulo, 2021.

Item	Before		After		p
	Median [IR]	Mean (sd)	Median (IR)	Mean (sd)	
Incidental recall		6.71 (1.684)		7.64 (1.393)	0.031
Immediate recall	9 [8.75–10]		10 [10–10]		0.02
Learning	9 [9–10]		10 [10–10]		0.059
Delayed recall	9 [7.75–10]		10 [10–10]		0.006
Recognition	10 [10–10]		10 [10–10]		0.564
Verbal fluency		20.14 (5.895)		21.93 (5.717)	0.425
Clock drawing	9 [9–10]		10 [9–10]		0.679
Total score		72.71 (8.801)		78.21 (6.192)	0.052

Abbreviations: IR: interquartile range; sd: standard deviation.

The MAC-Q score before and after the intervention is shown in Table 4. It is important to note that the assessment in this test is inversely proportional to the perception of memory. In the individual analysis by items of the instrument, the improvement is highlighted, when considering the median and statistical results before and after intervention to “Remembering the name of the person they just met,” “Remembering the telephone number you use at least once a week,” “Remembering where they put an object,” “Remembering news from a magazine article or television program,” and “In general, how would you describe your memory now compared to when you were 40 years old.”

**Table 4:** MAC-Q results before and after the intervention. São Paulo, 2021.

Item	Before		After		p
	Median [IR]	Mean (sd)	Median [IR]	Mean (sd)	
Remembering the name of a person they just met	4 [3–4]		3 [2–4]		0.016
Remembering the telephone number used at least once a week	3.50 [3–4.25]		3 [3–3.25]		0.039
Remembering where they put an object	4 [3–4]		3 [2–4]		0.018
Remembering news from a magazine article or television program	4 [3–4]		3 [2–3.25]		0.016
Remembering things that they intended to buy when they arrived at a store	4 [3–4]		3 [2–4]		0.058
In general, how would you describe your memory now compared to when you were 40 years old	8 [8–10]		8 [4–8.5]		0.023
Total score		26.43 (3.631)		21.36 (5.271)	0.002

Abbreviations: IR: interquartile range; sd: standard deviation.

Regarding the analysis of the qualitative data obtained from the interviews and the field diary, some notes by the researcher are presented in Chart 2. Participants discussed the impact of the group on their daily life, highlighting that this allowed them to diversify their routine, which had been restricted by the social restrictions imposed by the pandemic, as well as share their experiences with others.

**Chart 2:** Thematic analysis based on some of the researcher’s notes. São Paulo, 2021.

Thematic analysis		Transcription of participants’ speeches/performance observation
Benefits	Impact of the group on their daily life	P (1) “(...) you gave us the content with affection, it was very beautiful in this moment that we are in isolation, to feel this affection”. P (2) “(...) for those who are feeling depressed, this group soon morning can help”. P (9) “(...) despite having some things that we already used, it’s nice to know that it’s good, useful, that we’re on the right track and outside of the other things we had no idea about, like the strategy of using emotion to record information, this was new.”
	Socialization	P (12) “meeting new people from different universes was very gratifying (...)”. P (13): “This group is an opportunity for me to make friends and make plans for the future when we can meet in person”.
	Demystify beliefs	P (4) “(...) I always had trouble remembering the names of the people and I was a teacher, now I can see that it involves attention and the use of memory strategies”. P(7) “(...) I realize that when I demand control of everything, sleep is troubled, I accept the kind of life I lead today, I manage my life, I’m very dynamic... these are lessons I’m having with myself even, not to demand more from me than I am able to do (...)” P (14) “I always thought that the fact of making millions of things at the same time were positive, for me it broke this paradigm, I thought that it would save me from losing my memory.”
	Remote consultation	P (10) observed the optimization of the time and savings with transportation “(...) we are at home, it is economical and viable, you don’t need to take a car, bus and park”. P (9) “(...) it’s good that we start at ten o’clock and you can turn on the computer at five to ten, it’s not like it’s at USP that we have to leave the house well before”. P (12) was the only participant who reported having already participated in a face-to-face group with a similar proposal at his basic health unit of reference. It is there that he experienced a greater good among his participations” (...) so the group dispersed, out of affinity and empathy we approached to help with the coordinator’s guidance. What I feel in the virtual is that we are all seeing each other at the same time, there is a proximity, we are all interacting. At first I thought we would be further apart, on the contrary, I feel that the interest that leads to a good result.”
Difficulties	Device chosen for access	P (5): “(...) the cell phone limits more, the sound is not so good, the screen is small, the fact of being online challenges people of our age group who still are getting used to it”.
	Technology barriers/troubleshooting difficulty	There were interruptions that made it difficult to participate, these referred to the routine of the home, for example, family members passing by of the person and environmental noises such as those experienced by P (14) who was with works on the building and P (13) who lived near the airport. P (6), for example, could not turn on the microphone and, in an attempt to solve, entered and left the online room. There were, for example, episodes of problems with internet connection, interruptions while using the microphone when more than a person wanted to talk.

Abbreviations: P: participants (names in alphabetical order).

The elderly considered that the group favored their socialization, the sharing of daily experiences, and reduced feelings of loneliness. It also contributed to their following their own learning curve and a decrease in the constant comparison of current and past cognitive performance, which lowers anxiety and stress and results in greater acceptance of the changes inherent to aging.

The group helped demystify the belief that hyperstimulation and multiple simultaneous tasks are necessary to improve cognitive performance, especially with no concern about the quality of these stimuli. The relevance of selective attention and enjoyable activities to favor memorization was identified. Some of the older adults reported that understanding the complexity involved in the memorization process prompted them to increase their self-care and promoted healthy daily life habits. With respect to adopting shared group strategies, they cited learning new strategies, in addition to having used known mnemonic strategies more often.

When asked to reflect specifically on group remote consultation, they expressed surprise with the possibility of creating friendship bonds in a virtual setting. Advantages of this modality include being able to participate from anywhere with technological resources, lowering financial costs and time spent on urban travel. Also positive was the diminished risk of contagion, in addition to the more intimate online environment promoted by the group, such as “seeing pets and parts of the house.”

With respect to difficulties, since it is a distance meeting held in the house of each individual, complications arose that hindered participation. These include the domestic routine itself, such as family members walking behind the subject and environmental noise. Technological resources were also factors that interfered negatively in some moments of group development. For instance, internet connection problems, interruptions while using the microphone when more than one person wanted to talk, and the small screen size and difficulty visualizing the camera of all the participants when using a cell phone to access the platform. Thus, the type of device used to access the meetings was an important variable since, unlike the computer, a cell phone requires positioning the device, regulating the audio and microphone more accurately to make the sound clear and audible, as well as setting a grid view.

The participants felt that learning how to relate to the virtual medium was a valuable experience. In the beginning, they had more difficulty not interrupting others, but gradually organized themselves to contribute their impressions using chat tools and raising their hand. Some of the older adults required individual instruction to overcome their technological difficulties, as well as encourage family participation in the most difficult situations.

## DISCUSSION

This study aimed at analyzing effects of promoting cognitive health in a virtual group intervention for community-living older adults conducted during the COVID-19 pandemic. In agreement with what has been described in the literature, involvement in cognitively stimulating activities has shown to be promising in promoting cognitive health with aging^6,7^.

The frequency data on the mnemonic strategies most widely used after the group, namely, association, dual-task inhibition, increased attention, multiple coding, and categorization, corroborate a meta-analysis study that compared the efficacy of two modules of cognitive intervention, finding that multicomponent approach is more effective in creating transference/generalization for daily needs, because provide education on factors that affect memory, the use of memory strategies and support for the implementation of healthy lifestyle changes^24–26^. Another benefit identified in the group was cognitive performance assessed by applying BCSB before and after the intervention. Incidental, immediate, and delayed recall improved, corroborating the findings that cognitive plasticity extends to the end of adult life^25^.

Subjective memory complaints determined by MAC-Q also improved. It is presumed that mutual support among participants, interaction, and shared experiences of memory favored the decline in complaints. The literature reports that the group dynamic encourages the support of peers who are faced with similar challenges, resulting in older adults expressing fewer concerns about their memory^9^. The potential of a group to mitigate feelings of loneliness was also demonstrated. Interventions that promote behavioral changes in a group may achieve psychological well-being^27,28^.

An important issue to be addressed is that, although the participants defined themselves as cognitively healthy—through self-declaration both in the expression of interest form and in the researcher’s first phone contact—the older adults perceived themselves with subjective memory complaints. Also, the results showed positive changes in MAC-Q scores. In view of this, it would be possible to infer that the virtual group intervention could be an opportunity to identify community-living older adults with subjective memory complaints, which are not having specific monitoring by health professionals for this purpose. The subjective memory complaints are still poorly understood, but its importance is highlighted as it is a useful tool for detecting mild cognitive impairment or early Alzheimer’s disease^29^. Pereira et al addresses the need of primary care in assisting individuals with memory complaints^30^.

In this sense, the standardized virtual group intervention presented in this study, which aimed to promote cognitive health, can be a viable nonpharmacological intervention to be offered by primary health care teams, as it requires low-cost technology. In addition, as it is offered virtual, the intervention has the potential to reach a greater number of older adults as it does not require the availability of physical spaces in the health services, which is often an aspect that makes the implementation of interventions proposals difficult. In addition, virtual intervention has the potential to reach a greater number of people who reside in geographically distant territories or who face barriers to access services due to the difficulty in urban mobility. Added to this is the feasibility of offering the group in contexts that demand social distance, as was the case of the pandemic moment when restrictive measures were required.

It is worth adding that the group intervention presented in this study modified the subjective perception about the memory complaints of the elderly participants. It is considered that the emphasis of the intervention on comprehensive aspects such as promoting a healthy lifestyle, providing information on cognitive functioning in aging, managing emotions, demystifying cultural stereotypes related to the elderly, among other aspects may have favored the positive results found in this study. In accordance, Metternich et al found that expectancy change training (interventions that focus on cognitive restructuring and/or psychoeducation) positively influenced subjective memory complaints^31^.

Another result that needs to be addressed is that difficulties in handling technological resources interfered in group management. It was important for families to provide technological support, but primarily to teach older adults regarding the independent use through step-by-step instructions and individualized interventions. The older population can take advantage of telehealth services by overcoming their technical illiteracy barriers^28^.

In this respect, it was found that these strategies and technological experimentation during the course of the meetings gradually reduced the difficulties faced by the subjects. In addition, the older adults perceived that the group scenario was conducive to learning strategies that favored communication in a virtual medium. In this respect, despite the general group objective’s being linked to stimulating cognitive functions, a further benefit was the digital inclusion of older adults who had no previous technological knowledge.

American occupational therapists also found that technical problems related to the resources used were negative aspects of telehealth. However, despite these difficulties and the decrease in personal contact, positive aspects included increased access to care^18^. The older subjects of the present study also perceived that remote consultation is beneficial for the participation of individuals with difficult access, in addition to the reduced risk of contagion provided during the pandemic. Moreover, the decreased travel time and costs were also positive aspects.

A study found that most of the participants considered that telehealth should be a permanent intervention option, recommending its continued use after the epidemic^18^. The online format provides health services to older adults who otherwise would not receive them^17^. A review of health systems, public health departments, and senior centers also highlights that corroborating the study’s findings, there is growing evidence to suggest that healthy behaviors, such as being physically active, eating a healthy diet, and being socially involved, can promote cognitive health^32^.

The absence of a control group was a limitation of the study, due to the restriction of human resources available for the development of the research. Convenience sample was another limitation. It is suggested that future researches are conducted with control groups, as well as include heterogeneous samples in terms of age group and schooling, as well as subjects from the entire country, in order to analyze possible differences that interfere in memory performance, access to technology and making the best use of the group in a remote format.

This study demonstrated the viability of providing synchronous virtual group intervention for community-living older adults with focus on promoting cognitive health. There was a statistically significant improvement in memory, a reduction in subjective complaints and increased daily use of mnemonic strategies. We identified the therapeutic potential of the group to favor socialization during the pandemic and a decline in negative feelings such as anxiety and loneliness. Benefits were obtained from technological experimentation, primarily the digital inclusion of some of the older adults. The inference that this intervention could identify older adults with memory complaints that are not monitored by the health team is an important contribution of the study. Future interventions should apply specific tests on the participants to better identify those with risk of cognitive decline. Also, the study is relevant as it offers a model of intervention that can feasibly be applied by the primary health team, helping people who had difficulty to access the service.
